# A study of inclusive education provision in Zambia: Curriculum reform

**DOI:** 10.4102/ajod.v12i0.1067

**Published:** 2023-07-31

**Authors:** Mbulaheni O. Maguvhe, Allan Mutambo

**Affiliations:** 1 Department of Inclusive Education, School of Educational Studies, University of South Africa, Pretoria, South Africa

**Keywords:** inclusion, inclusive education, curriculum, curriculum reform, policy implementation, universal design for learning, Zambia, special education needs

## Abstract

**Background:**

The study is conducted to investigate whether curriculum reform for learners with special education needs (SEN) is taking place in Zambia.

**Objectives:**

The study objective were to investigate the extent to which curriculum had been reformed to facilitate the inclusion of children with SEN in Zambia; and determine stakeholders understanding of inclusive education policy, to evaluate the success of an inclusive programme in supporting the full inclusion of learners with SEN.

**Method:**

This study used a mixed method research design which involved data collection in seven provinces of Zambia. The researchers employed purposive sampling. The largest number of respondents were SEN teachers and administrators. The qualitative data collection tools included semi-structured interviews of individuals and focus groups. The quantitative data came from a questionnaire completed by teachers and supervisors as well as from government documents. The quantitative data were analysed using SOFA Statistics, while the qualitative data were analysed using ATLAS-TI 7.

**Results:**

Limited curriculum reform remains one of the main impediments to the implementation of the inclusive policy for children with SEN in Zambia.

**Conclusion:**

It is concluded that without curriculum reform the implementation of inclusive education in Zambia will be challenging.

**Contribution:**

There is a dearth of information regarding curriculum reform in Zambia. This is one of the studies that is attempting to plug the information gap on curriculum reform.

## Introduction

The inclusion of students with special education needs (SEN) continues to be a key issue in the quest for the provision of equitable education for all children (Naicker 2007; Theoharis & Causton 2014). This article is based on a study that investigated the extent to which inclusive education policy for children with disabilities had been implemented in Zambia since 1997.

The main focus areas of the study were: the understanding of inclusive policy by stakeholders; the success of the inclusive schools’ programme (INSPRO), curriculum reform to support the inclusion of learners with SEN, and the main impediments to the inclusion of children with SEN in Zambia. The article specifically focuses on the research question: To what extent has the curriculum in Zambian inclusive education schools been reformed to facilitate the inclusion of learners with SEN?

## Literature review

In Zambia, the three main educational principles according to Educating our Future SEN policy (1996:67) include a call for a continuum of placements for children with SEN, the existence of special schools and the provision of quality education. The above principles as outlined in the UNESCO (2008:3) definition of inclusion implies that successful and meaningful inclusion should be for the benefit of both those with disabilities and those without (Engelbrecht et al. 2016; Florian & Spratt 2013; Kauffman et al. 2018; Naicker 2018; Polat 2011).

### Current understandings and misunderstandings of what inclusive education should look like

Critics of inclusion continue to argue that the concept is a source of much confusion among scholars and other stakeholders alike. On one hand, other critics believe that inclusion, its goal and intervention models arise from the need to go beyond the physical integration of learners with SEN in regular education to the quality of educational outcomes (Hallahan, Kauffman & Pullen 2012; Warnock & Norwich 2010). On the other hand, scholars like Terzi (2014) is of the view that, for some learners, special education schools is one of the options to meet their needs. Cigman (2007) argues that children with SENs differ remarkably and ‘one size fits all’ principle should not be applied to them.

Some prominent detractors of inclusion such as Scruggs and Mastropieri (1996) and Kauffman et al. (2018) have continued to argue that most inclusive practices emphasise social integration to the detriment of the acquisition of content knowledge and skills when the goal of educational programming is not only socialisation but the attainment of meaningful outcomes for all learners. Similarly, Lindsay (2007:370) claims that inclusion should go beyond mainstream but should include meaningful participation in the education process. To this end, Lindsay (2007) proposes learners with special needs should be identified and provided for appropriately.

In addition, Florian and Spratt (2013:121) contend that using the inclusive pedagogy approach entails rejecting the labelling of learners based on ability. They also argue that diversity should be seen as a strength and not as a problem. Others suggest that the focus should be not only on the students with disabilities but on all students and then consider how to accommodate those with disabilities into mainstream education (Loreman et al. 2014; Naicker 2018).

**FIGURE 1:**
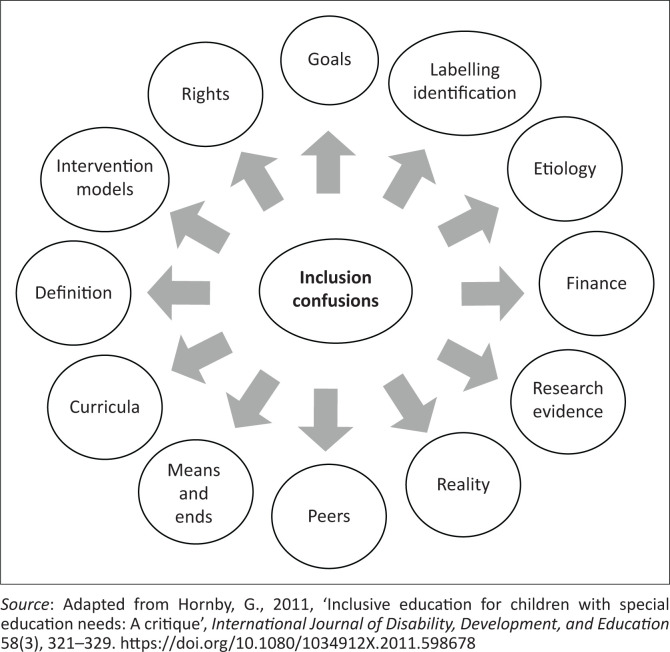
Issues relating to confusion with inclusion.

Hornby (2011) presents the sources of the confusion regarding inclusion and refers to these as ‘inclusion confusions’ (see Figure 1).

What Figure 1 depicts is that, if the implementers of inclusive education are still confused as highlighted by Hornby (2011) then diversity will be seen as a problem and not a strength. Confused implementers of inclusive education would still focus on students with disabilities and how to reasonably accommodate them in inclusive education settings (Hornby 2011). Instead, focus should be on all students with and without disabilities. For inclusive education to be effective, according to Hornby (2011), all things should be considered: (1) achievable inclusive education goals (among them not leaving any child behind), (2) identification of needs and support needed based on research evidence, (3) resources, (4) being realistic about what is possible and not possible.

### Curriculum reform

Curriculum reform to facilitate the inclusion of learners with SEN and other marginalised groups of learners generally accompanies many inclusive policy documents. In the Zambian case, item ‘vi’ (6) in ‘Educating our Future’ includes a call to address the need for curricula change to support learners with SEN (Republic of Zambia Ministry of Education 1996). However, how to develop pedagogy and curricula that will be inclusive and beneficial for all learners is easier said than done (Davis & Florian 2004:34). The need for curriculum reform is especially challenging because the focus on performance as a marker of success in mainstream education contexts persists. Wolfe and Hall (2003) have previously argued that inclusion of students with SEN necessarily requires redesigning of curriculum and the provision of appropriate classroom adaptations. These adaptations include, but not limited to: (1) seating arrangements, (2) rearrangement of the layout of the classroom, (3) limiting visual clutter on walls, (4) sensory stimulation with soft or noisy music, (5) reduction of noise in a classroom to accommodate learners with visual impairments, (6) controlling lighting to accommodate partially sighted learners and those with autism, (7) adapting furniture by lowering chairs or securing desks and creating slant boards throughout the classroom for writing support, and (8) adapting door handles for learners with orthopaedic impairments, cognitive and/or developmental delays.

A focus on improving the participation of learners with SEN is less appealing as they are considered to be a liability on high-stakes tests reports (Juneja 2018:21). Yuen et al. (2019) argue that a common curriculum following the principles of Universal Design for Learning (UDL) should allow for teachers to monitor the progress of all the learners. Hence, Flood and Banks (2021) suggest that UDL should not lose sight of the importance of monitoring learner outcomes. In this process, Mukminin et al. (2019) posit that this access to the curriculum by all learners should include those without SEN. However, it is worth noting, as Meier and Rossi (2020) argue, that UDL does not imply a ‘one size fits all’ curriculum document but a programme of learning which removes barriers to learning for all learners so that the curriculum becomes an integral part of the framework. In practice, Theoharis and Causton (2014) provide some practical steps and considerations to promote efficient and effective curriculum reform and implementation which are:

- buy-in from different stakeholders;
- understanding by school stakeholders, including parents, regarding curriculum changes;
- sufficient time for principals, teachers and parents to implement the new strategies required under the reform;
- adequate or effective allocation of resources for implementation;
- continued provision of professional development; and
- adequate efforts to build school capacity to implement school reform including: school stakeholder’s knowledge of roles and responsibilities, as well as stakeholder’s leadership and skills.

Curriculum reform has not been at the forefront in the quest for inclusive education. Although in the Zambian case, all curriculum documents after the first education reforms of 1997 have a specific section relating to equity and inclusion of learners with SEN albeit with no details about how to differentiate for exceptionality of learners with disabilities. In our view, education reform calls for a paradigm shift from separate standards, curricular and accountability systems between special education and mainstream education. It is therefore important, as Conn and Hutt (2020) posit, that all stakeholders are committed to the inclusion of all learners in the curriculum renewal and implementation. They argue that beneficence for all should be the goal through a curriculum that affords multiple pathways to learning. This includes the need for mainstream public schools to be conducive to SEN learners and that education must ensure equal access to training, skills development, equal opportunities as well as career paths of all learners.

## Theoretical framework

At the heart of this study is a focus on the development of children with SEN through the implementation of inclusive education policy in Zambia. This entails using a theoretical framework that can link the realms of philosophy, policy and practice (Houston 2017; Onwuegbuzie, Collins & Frels 2013). Bronfenbrenner (1992) believed that learning is a function of social interactions in a system of embedded structures (Lau & Ng 2014; Walker & Pattison 2016). In his earliest conceptualisation of the ecological system model, Bronfenbrenner presented four nested environmental levels in which a developing child exists and interacts with others – the microsystem, the mesosystem, the exosystem and the macrosystem (Bronfenbrenner 1979, 1992). To this end, Bronfenbrenner’s (1979) ecological systems model is used since it makes the best fit.

**FIGURE 2:**
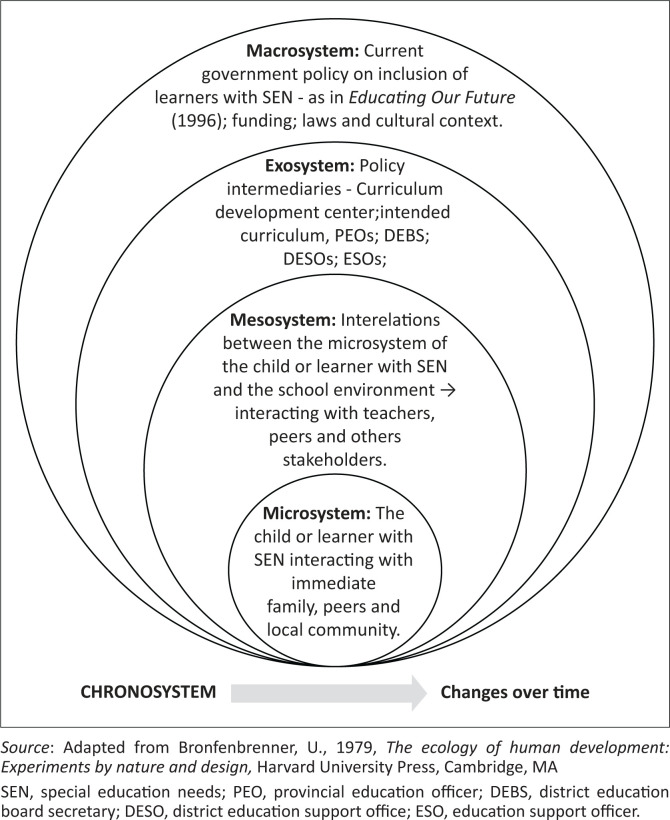
Ecological systems model applied to implementation of inclusive education policy for children with special education needs in Zambia.

Figure 2 shows how the ecological model is applied to the implementation of inclusive education policy in Zambia.

The focus on curriculum reform in Zambia entails that the teachers and school administrators in the mesosystem who are at the forefront of curriculum implementation, education standards officers (ESOs) in the exosystem, and policy initiators at the national government level in the macrosystem were targeted as participants and sources of relevant information for this study. The teachers interacting with learners and parents not only need a full grasp of the policy requirements but must have an inclusive curriculum that translates policy into actionable content and guidelines. Teachers will thereafter have the capacity to facilitate the participation of all the learners in inclusive education setting (Danforth & Naraian 2015; Spillane, Reiser & Reimer 2002; Stoll 2009).

To implement policy within the ecological systems approach, it is essential to take into account three interrelated aspects of capacity:

- Individual capacity (IC) implies one’s status or ability which is different from other peoples’ ability or potential.
- Collective capacity (CC) implies ways, means, processes and measures for people to work as a collective in schools, and
- Material Capacity (MC):MC is measured by amount of work completed or the quality of service rendered (Lasky 2005:4). This implies that for efficient and effective curriculum reform and implementation to occur, there must be mutual compatibility and linkages between various parts of the ecological system briefly explained above.

It seems teachers, learners, parents, government officials, administrators, etc., do not approach and embrace inclusive education from the same angle. It should be noted that in Figure 2, the model does not have concentric circles but has the circles touching each other. This implies that the sub-systems are connected albeit with varying degrees of familiarity (Neal & Neal 2013). The common centre of inclusive education according to the authors’ point of view is its implementation. It is advisable that all stakeholders should have common understanding of its challenges and how they should be addressed.

## Research methods and design

This article reports an aspect of a bigger study which employed a mix-method research design. To get a better understanding on how inclusive education is offered to learners with SEN, authors of this article employed a mixed-method design. Both qualitative and quantitative methods complemented each other during data collection (Maxwell 2013; Onwuegbuzie & Leech 2009; Onwuegbuzie, Leech & Collins 2010; Terrell 2012). Mixed methods helped researchers to gain a more complete picture than a stand-alone quantitative or qualitative study. The research design enabled researchers to integrate benefits of both the methods.

Data collection instruments included semi-structured interviews of both individuals and focus groups, observations, questionnaires and photographs. Other relevant data came from government policy documents, curriculum and instruction documents. The study was guided by Bronfenbrenner’s (1979, 1992) ecological system model in its analysis of findings. The framework has the capacity to link the realms of philosophy, policy and practice (Houston 2017; Onwuegbuzie et al. 2013). As applied to the study, the provision of equal education access to learners with SEN is a function of social interaction in a system of embedded structures (Lau & Ng 2014; Maxwell 2013; Onwuegbuzie & Leech 2009; Onwuegbuzie et al. 2010; Terrell 2012; Walker & Pattison 2016). For the purpose of this article, both qualitative data from individual and focus groups as well as document analysis, and quantitative data from questionnaires are presented.

### Sampling and participants

Purposive sampling was used to select a small group from a larger group with similar characteristics considered to be knowledgeable of, and informative about the phenomenon of interest; in this instance the provision of inclusive education to learners with SEN in Zambia (Maxwell 2013; Onwuegbuzie & Leech 2009; Onwuegbuzie et al. 2010; Terrell 2012). The study participants came from seven (Lua pula, Lusaka, Muchinga, Western, Southern, Central & Copperbelt) provinces of Zambia. A purposively sampled small group also participated in the quantitative part of this study. The aim was to gather valuable information and to assess the extent of the education reform. The target population which comprised of teachers, administrators and Education Support Officers was deemed to meet the criteria related to teaching learners with SEN, different provinces, gender, age, post description, responsibilities and duties. As a result, the largest number of respondents were SEN teachers and administrators currently involved in providing special education in Zambia.

There were seven focus group interviews which were held at seven different sites. The intention of the researchers was to interview 38 participants but because of circumstances beyond their control, they ended up interviewing 18 participants only.

### Data collection

#### Focus group interviews

Qualitative data through interviews were collected before quantitative data through questioners were collected. Participants were purposely selected and had similar characteristics including and not limited to knowledge of and information about curriculum reform and learners with special needs.

Each focus group comprised of three participants members plus the researcher. Each focus group interview took about 1h 30 min. Interviews took place at seven research sites (schools) and were conducted in English. This semi-structured interview focused on the six questions of the interview schedule. Listed below are the questions which were posed to focus groups:

- To what extent has the curriculum in Zambia been reformed to match key educational policy on SEN?
- What are some of the key reforms in the current curriculum that facilitate inclusion of learners with SEN?
- To what extent have curriculum reforms facilitating inclusion of learners with SEN been in line with the *Education Act 23, 2011* and the *Disability Act 6, 2012*?
- What would you consider the main impediments to curriculum reform and implementation targeting learners with SEN in Zambia?
- What aspects of curriculum reform with regard to SEN have successfully taken root?
- What feedback on curriculum reform with regard to SEN have you been getting from practitioners and other stakeholders?

#### Questionnaires

A purposive sample of 150 SEN teachers and 50 pre-service SEN teachers was anticipated. In four provinces, the questionnaires were delivered to the respondents by a proxy. All respondents who received the questionnaire had to complete and return back the consent forms prior data collection through both interviews and questionnaires. In two cases, the respondents were given the option to return the completed questionnaires in a self-addressed envelope provided by the researchers using a courier service. The statements used to collect data using questionnaires are:

- Statement 3: Has the curriculum in Zambia been reformed to facilitate inclusion of learners with SEN and learning disability (LD)?
- Statement 4: Is government education policy on SEN understood by the teachers and other stakeholders?
- Statement 8: What have been the main successes in implementation of inclusion of learners with SEN and LD in your school or centre?

### Document analysis

Zambia policy document on curriculum reform was downloaded from the Internet. In analysing document, special attention was given to units of meaning and set of categories. Researchers made notes from available documents. Researchers ensured authenticity by being open minded and referring to credible Zambian government documents. As part of document analysis, the following documents: Educating our Future (Republic of Zambia Ministry of Education 1996) and Zambia Education Curriculum Framework (Zambia MESVTEE 2013) were targeted.

Policy documents were analysed to determine whether issues of curriculum reform were taken into high esteem by the Government of Zambia. Furthermore, the researchers wanted to be sure whether those policies had clear inclusive education implementation plans.

### Data analysis

LeCompte (2000:146) asserts that analysis required turning data into results. As a result, research results cannot be accurate or reliable if pieces of data are incomplete or biased. Kothari (2004:122) suggests that data, after collection, be processed and analysed in accordance with the outline laid down for the very purpose of the research plan. Analysis also implies editing, coding, classification and tabulation of collected data.

Interview notes and questionnaires produced a large quantity of data that needed to be summarised and interpreted. Common threads, themes and, patterns were identified. Thematic data analysis for qualitative data was employed. The following thematic data steps were considered:

- Familiarisation
- Coding
- Generating initial themes
- Reviewing themes
- Defining and naming themes
- Writing up

In light of this idea, the researcher planned to be wholly immersed in the data so as to be familiar with the information or data and proceeded to systematically synthesise, organise, analyse, transcribe, segment and code data, eventually findings were then be presented. The researcher: (1) organised and prepared raw data for analysis, those were notes from archival documents and transcripts from interviews; (2) engaged or read data to get a general sense and reflected; (3) began by coding texts into consumable segments; (4) used codes to thematise or categorise for analysis; (5) presented how themes would help narrate a qualitative description; and lastly, (6) interpreted the collected data.

All 150 questionnaires for SEN teachers and 50 questionnaires for pre-service SEN teachers were received. Soon as data were collected, and while it was still fresh in the minds of researchers, the information was summarised and detailed notes were captured. Information for those notes included among other things time and date details, common themes or patterns, and any other unique observations. Researchers organised the data into different types such as, those being the observation notes, questionnaires and documents and/or artefacts. Questionnaires were also grouped according to who had completed them. This step also involved reading and re-reading the material (data) in its entirety, making notes of thoughts that sprang to mind and writing summaries of each transcript or piece of data that had been analysed. McMillan (2012:297) refers to this type of data as ‘etic data’ as they are representations of the researcher, whereas ‘emic data’ contain information provided by the participants in their own words. The objective with this step was to condense all of the information to key themes and topics that would provide some answers to the research question. The following quantitative data analysis steps were taken by researchers:

- Interrogate your question
- Cross tabulate quantitative results
- Expand with open-ended questions
- Analyse your open-ended data
- Visualise your results
- Interpret actionable insights

### Ethical considerations

The researchers adhered to research ethics requirements. During the interactions with participants, for instance, their rights, anonymity and confidentiality were protected (McMillan & Schumacher 2014). Participants names and the specific sites they are attached to are not directly mentioned in the study. This was to further guarantee the principle of anonymity and confidentiality. Codes in qualitative data analysis were also used. Nowhere in the research was mention made of participants’ real names and the sites they worked at. During the open discussions, the purpose of the study was thoroughly discussed with the participants and they were informed of their rights to terminate or withdraw from the study, if they so wished during the process of data collection (Booyse et al. 2001). The participants voluntarily accepted the participation by signing declaration forms that guaranteed the confidentiality and anonymity (ed. Delamont 2012). The University of South Africa, College of Education Research Ethics (2015/07/15/08904782/01/MC.) granted the researchers ethical approval.

## Results

### Qualitative data

#### Theme: Curriculum reform

The respondents were first asked to comment on the extent to which they think the curriculum had been reformed to facilitate inclusion of learners with SEN. The results from the ATLAS TI 7 report listing all the codes and documents indicate that respondents at all the seven sites where focus group interviews were conducted generally felt that there was a need for curriculum reform. Some respondents noted that the reform was not enough and presents with many challenges:

‘Unfortunately, the curriculum has not been reformed enough to include universal design for learning.’ (Participant 1, focus group 1, male)

‘As SEN teachers, we are the most challenged by the scanty content in the new curriculum because we have to try and modify the curriculum to include content for a variety of SENs.’ (Participant 2, focus group 1, female)

The responses from the two curriculum development specialists were more positive about curriculum reform, but they both conceded that there were some noted attempts of reform with resulting challenges:

‘I can say that attempts have been made to match the policy. However, this is not easy to cover all disabilities and SEN.’ (Participant 9, focus group 3, female)

‘There have been some changes to focus on knowledge value, skills and application for specific disabilities. This has covered mainly the visually impaired and hearing-impaired students.’ (Participant 13, focus group 5, male)

All the ESOs had similar concerns with the teachers. For example, an ESO declared:

‘We are using the same curriculum as the mainstream. So, what we do when the curriculum comes – we look at it and then ask what modification you can do as a school or as a teacher.’ (Participant 10, focus group 4, Male)

Another one commented:

‘Unfortunately, the curriculum has not matched the policy requirements. Teachers are expected to make modifications to the regular curriculum without the necessary materials.’ (Participant 15, focus group 6, female)

Similarly, another ESO mentioned:

‘However, there is not enough in the new curriculum framework to offer a guideline on how the strategies targeting learners with SEN, as presented in the policy, should be achieved.’ (Participant 13, focus group 5, male)

The responses from all the school heads (*N* = 8) also suggested a lot of pent-up frustration. For example:

‘There has been no curriculum reform to account for the diverse SENs. Asking the teachers to modify the content of the curriculum oversimplifies the problem.’ (Participant 11, focus group 4, male)

‘It is not enough to expect teachers to modify the curriculum. Modification not only requires training in SEN but experience in curriculum design.’ (Participant 18, focus group 7, male)

These comments indicate that the practitioners are generally very frustrated with the challenge of meeting the needs of learners with SEN when the Ministry of Education does not provide a universally designed curriculum (Ministry of Education 1977).

### Quantitative results

Quantitative data analysis is all about analysing number-based data (which includes categorical and numerical data) using various statistical techniques. The two main branches of statistics are descriptive statistics and inferential statistics (McMillan & Schumacher 2014).

In this study, descriptive analysis also known as descriptive analytics or descriptive statistics was used. It is the process of using statistical techniques to describe or summarise a set of data. Statements 3, 4 and 8 (Table 1 and Table 2) address the need for curriculum reform in line with the *Education Act 23, 2011* and the *Disability Act 6, 2012*.

**TABLE 1:** Frequencies report for statements 3, 4 & 8 with percentages.

Statement	Frequency	Column wise data %
**Statement 3**		
1.0	25	18.0
2.0	37	26.6
3.0	40	28.8
4.0	29	20.9
5.0	8	5.8
**Statement 4**		
1.0	20	14.4
2.0	14	10.1
3.0	21	15.1
4.0	34	24.5
5.0	50	36.0
**Statement 8**		
1.0	17	12.2
2.0	10	7.2
3.0	14	10.1
4.0	45	32.4
5.0	53	38.1

**Total**	**139**	**100.0**

*Source:* Mutambo, A., 2018, ‘A Study of Inclusive Education Provision for learners with Special Education Needs in Zambia: Policy Initiatives Perspective’, Unpublished D.Ed. thesis University of South Africa Pretoria

**TABLE 2:** Cross-tabulation report for statements 3, 4 & 8 against all locations.

Statement	Location													
	Central		Copperbelt		Eastern		Lusaka		Muchinga		Northern		Southern	
	Freq	Col %	Freq	Col %	Freq	Col %	Freq	Col %	Freq	Col %	Freq	Col %	Freq	Col %
**Statement 3**														
1.0	1	5.6	4	14.8	3	33.3	10	17.5	1	14.3	1	16.7	5	33.3
2.0	5	27.8	9	33.3	1	11.1	14	24.6	4	57.1	1	16.7	3	20.0
3.0	6	33.3	4	14.8	3	33.3	18	31.6	1	14.3	3	50.0	5	33.3
4.0	5	27.8	10	37.0	2	22.2	8	14.0	1	14.3	1	16.7	2	13.3
5.0	1	5.6	0	0.0	0	0.0	7	12.3	0	0.0	0	0.0	0	0.0
**Statement 4**														
1.0	3	16.7	4	14.8	2	22.2	8	14.0	0	0.0	0	0.0	3	20.0
2.0	1	5.6	2	7.4	2	22.2	4	7.0	2	28.6	1	16.7	2	13.3
3.0	1	5.6	6	22.2	2	22.2	9	15.8	0	0.0	1	16.7	2	13.3
4.0	7	38.9	6	22.2	1	11.1	13	22.8	2	28.6	1	16.7	4	26.7
5.0	6	33.3	9	33.3	2	22.2	23	40.4	3	42.9	3	50.0	4	26.7
**Statement 8**														
1.0	3	16.7	1	3.7	1	11.1	8	14.0	2	28.6	1	16.7	1	6.7
2.0	1	5.6	4	14.8	0	0.0	3	5.3	0	0.0	1	16.7	1	6.7
3.0	1	5.6	4	14.8	0	0.0	7	12.3	0	0.0	1	16.7	1	6.7
4.0	10	55.6	7	25.9	3	33.3	18	31.6	3	42.9	1	16.7	3	20.0
5.0	3	16.7	11	40.7	5	55.6	21	36.8	2	28.6	2	33.3	9	60.0

**Total**	**18**	**100.0**	**27**	**100.0**	**9**	**100.0**	**57**	**100.0**	**7**	**100.0**	**6**	**100.0**	**15**	**100.0**

*Source:* Mutambo, A., 2018, ‘A Study of Inclusive Education Provision for learners with Special Education Needs in Zambia: Policy Initiatives Perspective’, Unpublished D.Ed. thesis University of South Africa Pretoria

The statements are:

Statement 3: Has the curriculum in Zambia been reformed to facilitate inclusion of learners with SEN and LD?

Statement 4: Is government education policy on SEN understood by the teachers and other stakeholders?

Statement 8: What have been the main successes in implementation of inclusion of learners with SEN and LD in your school or centre?

The results for Statement 3 indicate that 44.6% disagreed that there had been sufficient curriculum reform as opposed to 26.6% who agreed and 28.8% opting for neutral. For Statement 4, 60.5% of the respondents agreed with the suggestion that curriculum had not been sufficiently reformed to account for the diverse needs of learners with SEN as opposed to 24.5% who disagreed with the statement. In response to Statement 8 which presents lack of curriculum reform as an impediment to the successful implementation of inclusive education for learners with SEN, 70.5% agreed with only 19.4% in disagreement and 10.1% opting for neutral. These results for Statements 4 and 8 suggest that the 28.8% neutral responses for Statement 3 might probably be leaning towards disagreement.

From the cross-tabulation results for Statements 3, 4 and 8 (Table 1 and Table 2) against all the locations, it appears that most of the respondents from the seven provinces sampled believed Zambia had seen limited curriculum reform to support the successful implementation of government policy on inclusion at the classroom level. The cross-tabulation results for Statements 3, 4 and 8 against the respondent’s current position indicate that respondents in all the current position categories generally disagreed with Statement 3, which suggests that there had been sufficient curriculum reform in line with the *Education Act 23, 2011* and the *Disability Act 6, 2012*. The highest disagreement rate was recorded by SEN lecturers and special education needs coordinators’ (SENCOs) with 66.7% and 55.5%, respectively. In the case of Statement 4, except for the guidance counsellors and teaching assistants, all the other respondent categories overwhelmingly felt that the curriculum had not been sufficiently reformed to account for the diverse needs of learners with SEN. This result is further upheld by the responses to Statement 8 which indicate that most of the respondents agreed that the lack of curriculum reform was one of the main impediments to successful SEN policy implementation in Zambia. The average rate of agreement for all groups was 73.0%.

#### Government documents analysis

Government and other official documents yielded important information that helped to complement or corroborate some aspects of data collected from other means. The main documents referred to were Education Sector: Implementation Framework 2008- 2010 Fifth National Development Plan (Ministry of Education 2007), Educating our Future (Republic of Zambia Ministry of Education 1996) and Zambia Education Curriculum Framework (Zambia MESVTEE 2013).

An inclusive education 3-year pilot project was initiated in Zambia in 2011. It was meant to strengthen national capacities for inclusive education. The project’s main objective was to improve access to quality education for children with visual impairments including those who were blind, those with low vision and refractive errors. It initially targeted 615 children and it was implemented in eight districts viz. Mufulira and Ndola districts of Copperbelt province and the Kazungula, Monze, Choma, Kalomo, Livingstone and Mazabuka districts of Southern province. The programme partners were the Ministry of Education, Zambia Open Community Schools, Child Hope, The Zambia Federation of Disability Organisations and Sightsavers.

It should be borne in mind that curriculum reform to facilitate the inclusion of learners with SEN and other marginalised groups of learners generally accompanies many inclusive policy documents. UNESCO (2008) makes a salient and prodigious call to address the need for curricula change to support learners with SEN. However, it remains difficult or tricky on how to develop pedagogy and curricula that will be inclusive and beneficial for all learners including those with SENs. Further, the Government Framework (Zambia MESVTEE 2013) indicates that implementation of inclusive education is inundated with a myriad of challenges including and not limited to: high enrolment levels, inadequate educational supplies, low staffing levels, inadequate classrooms and desks, dilapidated infrastructure, inadequate staff, teachers working under double or triple shift system, etc.

The Government of Zambia, in 2013, produced a document of the review of the curriculum which was titled the ZECF-2013. The Framework came with new dictates which included the introduction of early childhood education (ECE) into the mainstream Ministry of Education. What is worth noting is that the road map for the implementation of the New Curriculum shows that its implementation for ECE started in 2014. At that point in time, the education system in Zambia had been grappling with various problems. The ZECF-2013 was adding new responsibilities onto a system that was already having a lot of challenges particularly in the rural areas.

It was felt that the Zambian situation with its centralised nature of curriculum development led to a wide gap between the educational leaders on the one hand and the local community, teachers and students on the other. It is this very gap that makes it very necessary to constantly carry out studies on the curriculum after every review and/or reform to see how the curriculum implementation is fairing. Furthermore, the Framework focused on secondary schools leaving out the primary schools. The implementation of the reformed curriculum was exacerbated by the fact that it was for the first time in independent Zambia that the primary public schools have ECE as part of the systems.

Zambia’s Government Framework (Zambia MESVTEE 2013) posits that the philosophical rationale for educational provision is to nurture the holistic development of all individuals and to promote the social and economic welfare of society. This implies that the achievement of fairness in education should demand for educational policies which value and promote a multifaceted development of the learners, taking into account their uniqueness so that they can fully and rationally participate in the economic, cultural and social affairs of the nation.

In the process of document analysis of the above-mentioned framework, researchers discovered that though it does mention inclusive education, curriculum reform has not taken off the ground as anticipated. As alluded by participants above under qualitative data analysis, reasonable accommodation or inclusion is only practised by sensory impaired learners (visually impaired and auditory impaired) learners and not by other groups of learners with disabilities. Learners are still required to be taught by teachers who have special education qualifications. It is the researchers’ fervent view that if inclusive policies are formulated and proper structures are in place, inclusive education can be embraced and promoted.

## Discussion

The focus of this study is the development of children with SEN through the implementation of inclusive education policy in Zambia. This meant that the researchers used a theoretical framework that links the realms of philosophy, policy and practice. The researchers concur with Bronfenbrenner’s, 1979 and 1992 view that learning is a function of social interactions in a system of embedded structures.

The findings indicate that there has been very limited curriculum reform to facilitate the implementation of inclusive education policy as stated by participants under qualitative results above. The results from the questionnaire and interviews further show that what constitutes the ideal curriculum is not yet a reality in Zambia. Some proponents of inclusive education practice have argued that development of pedagogy and curricula that is inclusive is one of the main challenges of implementing inclusive education (Davis & Florian 2004; Naicker 2007; Theoharis & Causton 2014; Udvari-Solner & Thousand 1996; Wolfe & Hall 2003). This holds true in Zambia as most of the participants listed the limited curriculum reform to support inclusive practice as one of the impediments to inclusive policy implementation.

The most up-to-date curriculum document at the time of this study was the Zambia Education Curriculum Framework 2013 which, though it makes mention of the requirement of inclusion as part of educational policy in Zambia, does not provide practitioners with guidelines about how to ensure the inclusion of learners with SEN. Without a curriculum map that provides direction for teachers to provide a meaningful learning experience for all learners, the thrust for equity through inclusive practice in education will remain rhetoric (Flood & Banks 2021; Pugach et al. 2020; Theoharis & Causton 2014).

The curriculum implementers in Zambia should have a common understanding and work with a UDL framework to achieve effective inclusive education for all learners (Conn & Hutt 2020; Meier & Rossi 2020; Mukminin et al. 2019; Yuen et al. 2019).

### Limitations

There were several limiting factors that had a bearing on the validity and reliability of the findings of this research study. The first factor related to the reliance on a qualitative research design which is open to subjectivity. A mixed-method approach was used to mitigate the subjectivity by including quantitative data through administering a questionnaire that yielded quantitative data and qualitative data that were analysed using a statistical software programme. In the data analysis, findings from the qualitative data and quantitative data were put together to support finding claims and inferences. Moreover, data were collected using more than three data collection tools. Consequently, validity and reliability were enhanced through triangulation of the data collection instruments in the data analysis.

The second factor related to the use of purposive sampling and the sample size. Purposive sampling might not be representative of the population and the population members do not have an equal chance of participation as would have been the case if random sampling were used. Moreover, because the participants were somewhat homogeneous as educational practitioners the data loses a critical perspective. Another key point to note about the sample is that learners with SEN were not represented as a source of data. However, it is also important to note that the nature of data required to answer the research questions made it an imperative to target practitioners who could provide relevant responses because of their experience in education provision in general, and in the provision of SEN education in particular. In mitigation of the subjectivity, the participants were targeted from different sites in both rural and urban settings. Moreover, participation was open to all practitioners as long as they gave informed consent to participate.

The third factor related to the time available for data collection which was limited to about 12 weeks. This resulted in a smaller pool of possible participants being available. Consequently, less data was collected than anticipated, which affected the reliability of the findings. Having limited time also meant that the researchers could not reach all the ten provinces of Zambia. Nevertheless, seven out of ten provinces still represent 70% reach with respect to the original intent to cover all the ten provinces in the country.

The final factor related to the emphasis on qualitative data received from interviews. It is very plausible that some respondents might have provided positive responses to avoid upsetting the status quo. However, the assurance of anonymity and confidentiality seemed to have mitigated this concern based on the patterns arising from the data analysis.

### Recommendations

Firstly, it is imperative that the curriculum is reformed to come up with a UDL to facilitate the efficient and effective inclusion of learners with SEN in the mainstream classrooms. To this end, research-based curriculum reform should be accompanied by teacher capacity building initiatives and processes for the implementation of inclusive curriculum. It is important to note that starting at pre-knowledge in this effort and building up from there is essential for effective implementation of inclusive curriculum.

Secondly, there is a need for specific guidelines for curriculum implementation involving differentiation for the diverse needs of all learners. It is recommended that the curriculum should come with SMART objectives that are specific, measurable, achievable, realistic and are time-bound. This would allow for evaluation of inclusive curriculum implementation. Moreover, there must be clear standards for learner progress in the curriculum and that will only be possible through rigorous curriculum reform in Zambia.

Thirdly, it is recommended that there should be both support instruments and evaluative instruments at the disposal of education standard officers and other policy intermediaries to build in continuous quality assurance and quality improvement. Reflective practice is required to enhance efficient and effective inclusive curriculum implementation.

Finally, curriculum implementation will require adequate resourcing and infrastructure from government and other stakeholders in education. This calls for adequate government funding and investment in education. Construction of more classroom space, training of more special education specialists and paraprofessionals to support the inclusion of learners with SEN must go along with efforts at implementing UDL principles and practices.

## Conclusion

This article suggests that a lack of meaningful curriculum reform is a major challenge that efforts at curriculum implementation guidelines development will face in the Republic of Zambia. To achieve curriculum implementation, the whole educational system should be made ready to contribute to the common goal to achieve the goals of inclusive education provision in Zambia. Whether it takes the form of curriculum reform or renewal, the key lies in the fact that all requisites for the efficient and effective implementation of inclusive education are interrelated and interconnected.
